# Short and long terms healing of the experimentally transverse sectioned tendon in rabbits

**DOI:** 10.1186/1758-2555-4-14

**Published:** 2012-04-26

**Authors:** Ahmad Oryan, Ali Moshiri, Abdul-Hamid Meimandi-Parizi

**Affiliations:** 1 Department of Pathobiology, School of Veterinary Medicine, Shiraz University, Shiraz, Iran; 2 Department of Surgery and Radiology, Group of Clinical Studies, School of Veterinary Medicine, Shiraz University, Shiraz, Iran

**Keywords:** Tendon healing, Rabbit, Surgical repair, Ultrastructure, Biomechanics

## Abstract

**Background:**

The incidences of tendon injuries in certain sections of human or animal populations such as athletes are high, but every human or animal, regardless of age or level of activity experiences some degree of tendon injury. In spite of the various investigations of injuries and treatment, comprehensive studies dealing with the histological, ultrastructural and biomechanical aspects of healing of load-bearing tendons are rare. This study was designed to compare the outcome of healing of the transverse sectioned superficial digital flexor tendon (SDFT) after 28 and 84 days post injury (DPI) in rabbits.

**Methods:**

Forty white New Zealand mature female rabbits were randomly divided into two equal groups of 28 and 84 DPI After tenotomy and surgical repair of the left SDFT, the injured legs were casted for 14 days. The weight of the animals, tendon diameter, and clinical, radiographic and ultrasonographic evaluations were conducted at weekly intervals. The animals were euthanized on 28 and 84 DPI and the tendons were evaluated for histopathological, ultrastructural, biomechanical and percentage dry weight parameters.

**Results:**

Although the clinical, ultrastructural, morphological and biomechanical properties of the injured tendons on day 84 showed a significant improvement compared to those of the 28 DPI, these parameters were still significantly inferior to their normal contra-lateral tendons.

**Conclusions:**

This study showed that tendon healing is very slow and at 84 days post-injury the morphological and biomechanical parameters were still inferior to the normal tendons and many collagen fibrils still had the same diameter as those seen at 28 DPI.

## Background

Tendon injuries present a significant clinical challenge to orthopedic surgeons and are a major problem in sports and occupational medicine [1-5]. This unique tissue plays an essential role in the biomechanical function of the musculoskeletal system by stabilizing and guiding the motion of diarthrodial joints [5-9]. Tendon is susceptible to both excessive tensile loads and compressive forces [2,10,11]. Injury represents a failure of the cell matrix to adapt to load exposure, which can be either acute or secondary to cyclic overuse [3-5,12].

A healthy human tendon does not rupture accidentally [13-16], however, in cases of trauma, during surgery and similar conditions, it can be transected by a sharp instrument [12-14]. There are many traumatic cases in veterinary medicine such as car accidents, hitting and other related conditions in which sharp and hard metal materials penetrate the skin and cut the intact tendons, resulting in tendon rupture [2,10,13]. In another approach, in such cases of orthopedic surgery, for example, the internal fixation techniques to reduce fractures of the tibia or metatarsal bones, the surgeon should expose the fractured site in a manner that allows the fixation plate or other fixation materials to be implanted, implantation of the screws to be facilitated and the anatomical reduction of the fractured site to be achieved. During these procedures it is quite common to cut the intact tendons [13,14].

Tendon ruptures are initially treated by direct suturing techniques as a standard of care [10,12,13,17]. The suture technique is of primary importance in providing a stiff and strong repair throughout the early healing interval [10,14,18,19]. However, despite improved surgical techniques and advances in rehabilitation techniques, early complications such as rupture of the repair site and restrictive postoperative adhesions are still encountered [2,3,20-22]. A repaired tendon needs to be protected for weeks until it gains enough strength to handle physiological loads [23]. Thus, surgical results are unpredictable, with pre-injury functional levels difficult to attain [3,13,20-22].

With these challenges the knowledge of healing concepts in the acute transverse section in the experimental model are still unclear. Thus, it is apparent that a better understanding of the function of tendons, together with a knowledge of their biology and healing potential is necessary for investigators to develop novel strategies to accelerate and improve the process of healing of transverse sectioned tendons [4,14].

The present study was designed to investigate the outcome of the healing response of two phases of early and late tendon healing to determine the effect of time on the biomechanical and morphological characteristics of the transverse sectioned superficial digital flexor tendon after primary repair and balanced post-operative rehabilitation in rabbits. The authors basic hypothesis was that the healing of the experimentally induced transverse sectioned of the SDFT, similar to other types of tendon injuries such as tendinopathy, is slow and the structural and functional properties of the injured tendon will somehow improve during the later stages of healing. However, the structural and physical performance of the injured tendon is significantly inferior to that of the normal contralateral tendon several months or even years after tendon injury. The collagen fibrils are still immature and the biomechanical performance is significantly inferior to the intact tendons, even for a long time after injury.

## Materials and methods

Type of experimental study: simple accidental interventional study.

### Animals

Forty skeletally mature, 10-14 month-old, female white New Zealand rabbits of 1.66 ± 0.76 kg body weight were randomly divided into two equal groups and evaluated on 28 and 84 DPI. They were kept in individual cages at 25°C, 60% humidity and were maintained on the same standard rabbit diet with no limitation of access to food or water. Each animal served as its own control and the right SDFT was used as its normal control.

### Injury induction

**Figure 1:**
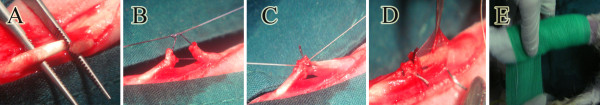
**surgical method: A**) Injury induction. **B**) Modified Kessler core suture. **C**) Running pattern. **D**) Sheet closure. **E**) Immobilization technique.

The animals were anesthetized by intramuscular injection of 3 mg/kg Xylasin as a premedication and 30 mg/kg Ketamin HCl for anesthesia. The left hind leg of each animal was designated as "experimental" and the skin over the common calcaneal tendon (CCT) was shaved and disinfected, using normal surgical aseptic technique. A 2 cm longitudinal incision was made through the skin and subcutaneous tissue, approximately 0.5 cm distal to the gastrocnemius muscle and 0.5 cm above the calcaneal tuberosity (CT), and the CCT complex was exposed [7,8,12,17,24]. An incision was made in the paratenon; the SDFT was exposed and carefully dissected. The SDFT was completely incised transversely at the mid part of the tendon, approximately 1.5 cm distal to the gastrocnemius muscle and 1.5 cm above the CT (Figure 1A). Immediately after tenotomy, the tendon proper was sutured using the modified Kessler technique [23,24] with absorbable polyfilament polygalactin 910, 4-0 sutures (Ethicon coated Vicryl, taper cut needle, Johnson & Johnson, Trademark, USA) (Figure 1B). The edges of the tendon were sutured by running pattern, with the same material no. 6-0 (Figure 1C) [9; 12, 17]. The paratenon and the skin were routinely sutured (Figure 1D). Four knots in all patterns was applied for all of the animals. After surgery, a cast (Dyna cast 5 cm, An-Yang, Korea CO., LTD) was applied for two weeks at 70° extension (Figure 1E).

### The pre euthanasia measurements

Before injury induction, the animals were weighed and the diameter of the right and left tendons and the covering skin were blindly measured as an index of the tendon swelling and post-surgical inflammation. The tendon and the covering skin diameter around the injury site, with a comparable area of the uninjured contra-lateral tendon, were measured using a micrometer measurement device (Samsung, Seocho-gu, Seoul, Korea). The weight of the animals and the tendon diameter were measured and analyzed before injury, and then at weekly intervals until the animals were euthanized. Each measurement was made three times to ensure that the repeatability of the measurements of the width was within 0.2 mm. From these, the average cross-sectional area of the tendon, together with the fascia and skin over it was calculated.

For the clinical investigations, two observers blindly determined the lameness and weight bearing capacity of the rabbits. The walking activity of each animal in the cage was checked 3 times a day (8 h intervals). The assessment was qualitative. Lameness and comfortable/uncomfortable physical activities were defined as tarsal flexion degree of each animal, both in the cage and on the floor, weight distribution of each animal on the hind limbs, both in the cage and on the floor, pain in palpation of the injured area, pain in complete extension of the hind paw and toe, and heel position of the injured leg [12].

The radiographic and ultrasonographic observations were blindly evaluated by a radiologist at weekly intervals for 12 weeks to define whether the tendon injury had altered the joints and bony structures of the hind paw. In addition, the maturity of the animals was confirmed by radiology. All animals were found to be mature. Lateral and dorsoventral position radiographs were provided from the whole body, using large film at 80 KVp and 6 MA. The cross-section echo texture of the SDFT of the rabbits due to their low diameter and view was not diagnostic; therefore, the animals were sonographed at longitudinal section with a 12 MHz linear probe (Simense SLR-400, Berlin, Germany; Echowave 3.23 software). The authors considered the following criteria to define the differences in the injured tendons with those of their normal contra-lateral ones: A) The ultra sonographical echogenicity of the tendon: 1) hyperechogenicity, 2) hypoechogenicity; B) The relation of the hyperechogenic area of the echotexture tendons compared to the hypoechogenic area of the echotexture tendons: 1) the tendon had a smooth echogenicty. This means that there was no diagnostic hyperechogenicity besides that of hypoechogenicty, 2) the tendon had no smooth echogenicity. This means that there were areas of hyperechogenicity besides those of hypoechogenicity known as an amputated view; C) The movement of the tendon with finger in ultrasonography: 1) The SDFT could be well moved transversely, 2) The SDFT movement in a transverse direction was not diagnostic; D) The diameter of the SDFT was calculated using the scale of the ultrasonography machine: 1) Thick, 2) Medium, 3) Thin [12,17].

### Ethics and euthanasia

Twenty eight and 84 days after injury induction, the animals were euthanized by Na-thiopental (50 mg/Kg), Xylazin (20 mg/Kg) and Ketamin HCl (300 mg/Kg). The study was approved by the local ethics committee of our faculty, in accordance with the ethics standards of “Principles of Laboratory Animal Care”.

### Sample collection

The specimens from each of the injured and uninjured SDFT of ten of the animals of each group were longitudinally sectioned in three pieces for light and electron microscopic studies and percentage dry weight analysis. In the remaining ten animals of each group, both injured and contra-lateral SDFT were carefully dissected from the surrounding tissues for biomechanical testing. The SDFT was cut and separated proximally to include 3 cm of the muscle belly and distally to the site of insertion of each phalangeal branch [7,9,25,26].

### Light microscopy

After fixation in 10% neutral buffered formalin, the tendon samples were washed, dehydrated, cleared, embedded in paraffin, sectioned at 4–5 μm, stained with haematoxylin and eosin and examined by a light microscope (Olympus, Tokyo, Japan). The cells and vascular populations of each section were estimated using an eye piece graticule. An average was then taken from five different microscopic fields for each cell type. Duplicate counts were carried out by double blind method. In addition, using a digital camera (Sony, T-700, Tokyo, Japan), the pictures from each slide were transferred to a computer for morphometric analysis. Maturity of the tenoblasts together with the density of the collagen fibers and blood vessels on the normal and inverted photomicrographs were determined using Adobe Photoshop cs-3 10 final [12]. The mesenchymal cells at the injury site were divided into three categories based on their diameter, cytoplasmic granules and cell staining capacity. The largest elliptical cells with high granular and basophilic cytoplasm were determined as immature tenoblasts (fibroblasts). The long, cigar-shaped cells with less granulated but eosinophilic cytoplasm were estimated as tenocytes, while the medium sized cells with neutral cytoplasm and medium amounts of cytoplasmic granules were accounted as mature tenoblasts (fibroblast) [12]. Additionally, the crimp pattern, tissue maturation, alignment and density, together with the types of degeneration and foreign body reactions on each sample, were qualitatively and semi-quantitatively analyzed and scored. The number of vessels was evaluated in 5 fields of each histopathologic section with x200 magnification. The mean of the data for each animal and the mean of the histopathologic sections of the animals of each group were then determined [26].

### Electron microscopy

The samples from the injured site and a comparable area of the normal contra-lateral tendon were fixed in cold 4% glutaraldehyde, dehydrated and embedded in Epon resin 812. Thin sections of 80-90 nm in diameter were cut and standard methods were employed for production of the transmission electron micrographs (Philips CM 10 transmission electron microscope, Eindhoven, Netherlands) [26]. Ultra-micrographs of different final magnifications (5,200-158,000) were taken for studying the collagen and elastic fibrillar morphology, inflammatory cell constituents and tenoblast’s maturity. For fibrillar density, ten pictures were captured from ten horizontal and vertical fields; for each sample the surface area of the collagen fibrils regarding their category dependency were measured and analyzed. The number of collagen fibrils and their diameters in five different fields of each tissue section was measured. The collagen fibrils were divided, based on their diameter, into 5 different categories of 33-64, 65-102, 103-153, 154-256 and 257-307 nm (nm) respectively [12]. The number and diameter of the collagen fibrils were measured, and their mean diameter was calculated by a computerized morphometric technique, using Adobe Photoshop CS4. In addition, the number of elastic fibers of each field was counted and their maturity was qualitatively evaluated.

### Biomechanics

After application of the standard preservation methods, biomechanical tests were performed using a tensile testing machine (Instron Tensile Testing Machine, London, U.K.) [12]. The specimens were mounted between the two metal clamps and were subjected to tensile deformation at a strain rate of 10 mm/min and the load deformation and stress–strain curves were recorded by a personal computer. The complete method has previously been described [25]. The ultimate tensile strength, yield strength, ultimate strain, yield stain, stress and stiffness were determined.

### Percentage dry weight

The samples were weighed immediately after euthanasia and were freeze-dried (Helosicc, London, UK) to a constant dry weight as previously described [9,12].

### Statistical analysis

After application of the normality distribution test, the injured tendon of each animal was compared with the normal contra-lateral tendon of the same animal, using paired sample *t*-Test. The right and left tendons of the 28 DPI animals were compared with the right and left tendons of the 84 DPI animals, using the independent sample *t*-Test. Nonparametric tests were applied to check the results again. Statistics were performed using the computer software SPSS version 17 for windows (SPSS Inc., Chicago, IL, USA). Differences of *p < 0.05* were considered significant [9,17].

## Results

### Clinical and gross morphologic findings

**Figure 2:**
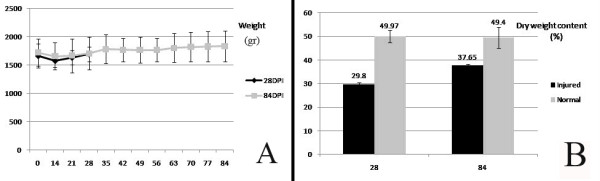
A) Weight of the animals. B) Dry weight content of the injured and normal tendon of both groups.

**Figure 3:**
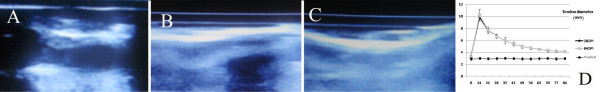
**Ultrasonography: A)** Injured tendon on 28 DPI: The diameter of the tendon is greatly increased; however, it is not uniform and shows amputated view. Its echogenicity is not detectable from the surrounding fascia. **B**) 84 DPI: Injured treated tendon of an 84 DPI rabbit. The diameter of the tendon is comparable to normal tendon. The tendon proper is detectable from its surrounding fascia. **C**) Normal contra-lateral tendon. The diameter of the tendon is lower than A and B. No amputated view is present and the echogenicity is smooth. **D**) The tendon diameter of injured tendon significantly reduced from day 21 to 84 post- injury.

There were no significant differences in the weight in any of the 12 week intervals and the animals of both groups had almost normal weight gain during the course of the experiment (Figure 2A). The diameters of the injured tendons were significantly larger than those of their uninjured normal contra-laterals at 14, 21 and 28 days post-injury (*P < 0.0001*). Although the degree of swelling gradually subsided after 28 days, at the end of the experiment the diameter of the injured tendon was still larger than the uninjured normal contra-lateral ones (*P = 0.001*) (Figure 3D).

In the first two weeks post-injury, the animals showed lameness with lower amounts of physical activity and the injured area was hyperemic and warm. From day 14 post-injury these abnormalities gradually started to relieve so that the physical activity of the animals returned back to an almost normal level on day 35 post-injury. At this stage, the hyperemia was resolved and the incision site on the skin was sealed with new granulation tissue. At five weeks post injury the weight bearing capacity on the injured leg was comparable to the normal contra-lateral leg.

No lesions such as soft tissue swelling, calcification, osteoarthritis, bone fracture and abnormal signs of radiolucency or radio opacity were observed in the radiographs of either group at any post-surgical interval.

Due to the small diameter of the SDFT, there was no diagnostic imaging at cross sectional ultrasonography, but the longitudinal sections at ultrasonographic levels, were diagnostic, in order to compare the two stages with the normal tendon. Amputated view with irregular echogenicity in the lesions was evident in the first 6 weeks post injury. However, from day 49 up to the end of the experiment, there was no evidence of amputated view and the echogenicity of the injured tendon proper showed considerable improvement; however, at this stage they were still inferior to their normal contra-lateral tendons (Figure 3A-C).

**Figure 4:**
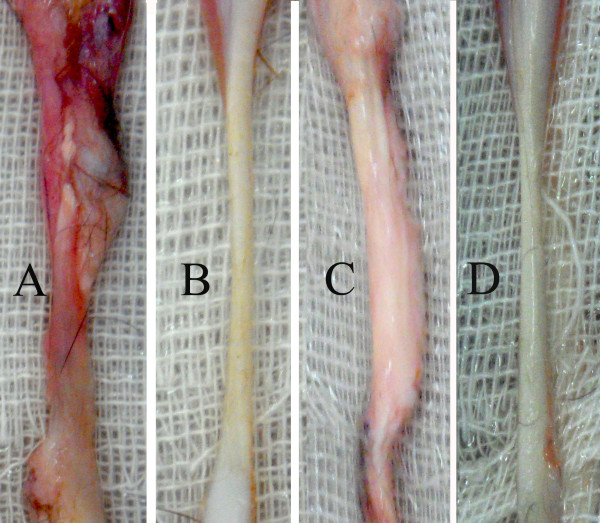
**Gross pathology: A)** Injured tendon of a rabbit of 28 DPI showing swelling, hyperemia and peritendinous adhesion. It is much thicker than the normal contralateral tendon. **B**) Normal tendon of a 28 DPI rabbit having shiny and normal appearance. **C**) Injured tendon of 84 DPI group. The hyperemia has disappeared, the tendon diameter is slightly reduced and no adhesion is seen in the peritendinous area **D**) Normal tendon of a rabbit of the 84 DPI group.

As shown in Figure 4, there were no signs of gap formation and dehiscence in the injured area and the scar tissue was formed around the suture material on day 28 post injury. At this stage, the lesions were severely hyperemic and adhesion with loose areolar connective tissue surrounded the entire injured area. However, at day 84 post-injury, the suture material disappeared and the surface of the injured area was smoother and aligned. At this stage, the tissue adhesion to the surrounding areas, and hyperemia was reduced, and the tendon showed a glistening and fibrotic appearance. However, there were still diagnostic differences between the injured tendons with those of the normal contra-lateral ones at this stage of healing (Figure 4).

### Histopathology

The uninjured contra-lateral tendons exhibited parallel bundles of collagen fibers and aligned tenocytes. The cells had dark spindle-shaped nuclei with small amounts of eosinophilic cytoplasm. In the longitudinal section the collagen fibers displayed a characteristic "crimp" pattern.

The injured area at day 28 post-injury was edematous and hypercellular and high degrees of cellularity consisting of fibroblasts, lymphocytes, plasma cells and macrophages were present in their lesions. The cells and collagen fibers showed no sign of crimp pattern and were arranged in a random orientation, resulting in peritendinous adhesion. The remnant of the suture material was still present in the lesions. The new reparative cells had pale vesicular nuclei and were larger than normal mature tenocytes. The cytoplasm of the regenerative tenoblasts was basophilic in contrast to that of the mature tenocytes. Numerous capillaries were evident in the lesions at this stage.

**Figure 5:**
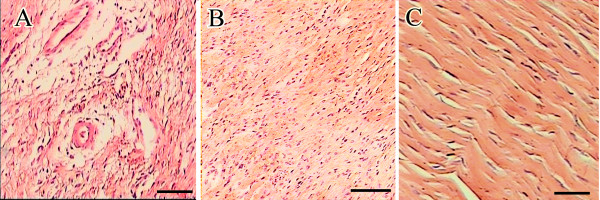
**Histopathological findings: A**) Tissue section from the injured area of a 28 DPI rabbit. The tissue is disorganized and edematous. Edema is not only conspicuous in the tendon proper, it is quite prominent in the perivascular area. Few inflammatory cells are seen in the section. **B**) Tissue section from the injured area of an 84 DPI rabbit. The tissue is still hypercellular, however, the fibroblasts and collagen fibers show a linear alignment. **C**) Tissue section from a normal tendon of a rabbit of 28 DPI. The collagen fibers and tenocytes are arranged along the longitudinal axis of the tendon and show a crimp pattern. The tenocytes have cigar shaped nuclei (Scale bar = 56 Mm).

The injured area at day 84 post-injury showed a proper alignment of tenoblasts, mature tenocytes and collagen fibers. The tissue density and crimp pattern of the lesions were more improved and were almost comparable to those of the normal contra-lateral tendons. Compared to those of the 28 days post-injury, fewer lymphocytes, plasma cells and macrophages were seen in the lesions of these rabbits. The maturity of the tenoblasts and numbers of the tenocytes of the lesions were significantly higher on day 84 compared to those of the 28 days post-injury respectively (*P = 0.012*, *P = 0.009*). The number of blood vessels was fewer and their caliber was larger than those of 28 days post-injury (Table 1) (Figure 5).

**Table 1:** Histopathology: number of cells with their differentiation and vascularity (mean and Standard deviation), (28 DPI vs. 84 DPI vs. their normal contralateral).

	Four weeks post injury		Twelve weeks post injury	
Variable	Injured tendon	Normal tendon	Injured tendon	Normal tendon
Fibroblast	237.75 ± 26.58	6.00 ± 12.00	211 ± 22.22	33.00 ± 49.65
Fibrocyte	Negative	29.00 ± 7.52	14.00 ± 7.43	34.25 ± 12.03
Macrophage	6.50 ± 1.64	Negative	3.25 ± 1.50	Negative
Lymphocyte	15.75 ± 7.84	Negative	12.25 ± 3.68	Negative
Neutrophil	Negative	Negative	Negative	Negative
Mature fibroblast	8.50 ± 2.51	Negative	15.50 ± 3.00	Negative
Immature fibroblast	28.25 ± 5.56	Negative	15.00 ± 4.83	Negative
Vascularity	3.75 ± 3.30	Negative	2.00 ± 1.63	Negative

Number of histopathologic slides for each group = 10, number of histopathologic field = 5, pair sample *t-Test* and independent sample *t-Test (P < 0.05)*, magnification for tenoblasts, tenocytes, macrophage, lymphocyte and neutrophil = x200 and for mature and immature tenoblasts (fibroblasts) and vascularity = x800.

### Electron microscopy

As shown in Table 2, five different diameters of collagen fibrils were observed in the transverse section of the uninjured contralateral tendons of 28 and 84 days post-injury rabbits. However, the proportion of the large collagen fibrils, in the range of 256-307 nm in the normal contra-lateral tendons of the animals of the 84 days post-injury lesions, possibly due to carrying more weight for a longer time, was significantly greater (*P = 0.001*) than the 28 days post-injury ones. The normal contralateral tendons on day 84 contained significantly larger collagen fibril (256-307 nm) (*P = 0.001*). In addition, the number of collagen fibrils in the range of 65-102 nm were also significantly more than those of the normal contralateral tendons on day 28 post injury (*P = 0.001*).

**Table 2:** Ultrastructural fibrillar count and morphometric analysis of the injured tendons and their normal contralateral tendons on days 28 and 84 post injury and surgical intervention

	Variable	Four weeks post injury		Twelve weeks post injury	
		Injured tendon	Normal tendon	Injured tendon	Normal tendon
*Number* of collagen fibrils at different range of collagen fibrils diameter	33-64 nm	617.50 ± 91.61	49.75 ± 6.13	378.00 ± 51.55	43.25 ± 7.36
	65-102 nm	Negative	9.50 ± 2.08	88.00 ± 25.85	17.50 ± 1.29
	103-153 nm	Negative	12.25 ± 2.50	Negative	14.50 ± 2.08
	154-256 nm	Negative	20.25 ± 2.21	Negative	10.00 ± 1.41
	257-307 nm	Negative	Negative	Negative	8.50 ± 0.577
	Total	617.50 ± 91.61	91.75 ± 1.70	466.00 ± 67.14	92.75 ± 8.65
Diameter of collagen fibrils at different range of collagen fibrils diameter					
	33-64 nm	37.07 ± 4.75	38.20 ± 4.33	46.79 ± 4.37	37.73 ± 2.25
	65-102 nm	Negative	98.51 ± 2.65	70.50 ± 4.91	98.61 ± 3.04
	103-153 nm	Negative	149.56 ± 1.89	Negative	152.12 ± 0.74
	154-256 nm	Negative	234.39 ± 8.13	Negative	236.55 ± 9.07
	257-307 nm	Negative	Negative	Negative	278.28 ± 4.73
	Total	37.07 ± 4.75	177.50 ± 15.71	51.12 ± 4.11	193.25 ± 2.50
Number of elastic fibers					
	Elastic fibers	0.50 ± 0.57	Negative	0.50 ± 1.00	Negative
Collagen fibrils/Area^2^					
	Density	51.67 ± 9.50	94.59 ± 3.00	84.56 ± 3.16	95.66 ± 2.77

Number of tissue section for each group = 10, number of ultra-micrograph in each tissue section = 5, pair sample *t-Test* and independent sample *t-Test (P < 0.05),* magnification = x39000. The collagen fibrils based on their diameter in the normal tendons were divided into 5 categories and the number of collagen fibrils and their diameter in each category were analyzed. Each data is the mean of the 50 ultra-micrographs. Normal contra lateral tendons contained 5 different ranges of collagen fibrils based on their diameter from 33 to 307 nm. The injured tendons on day 28 after injury had collagen fibrils in the range of 33-64 nm and the injured tendons on day 84 after injury had collagen fibrils in the range of 33-102 nm. The differences between groups were significant (P < 0.05). Number of the elastic fibers was not significant between groups (P > 0.05) and the collagen fibrils density (transvers area of the fibrils in the ultra-micrographs) was significantly different between groups (P < 0.05) and the injured tendons did not gain the normal density after 28 and 84 days post injury and surgical intervention.

The injured tendons of the 28 days post-injury consisted of a homogenous population of small-sized, new, regenerated, haphazardly organized fibrils of 33-64 nm, and mean diameter of 37.07 ± 4.75 nm, which made up all of the tendon bulk. The maximum diameter of the collagen fibrils at the injured site of the 28 days post-injury lesions was 15.8% of those of their uninjured normal contra-lateral tendons.

**Figure 6:**
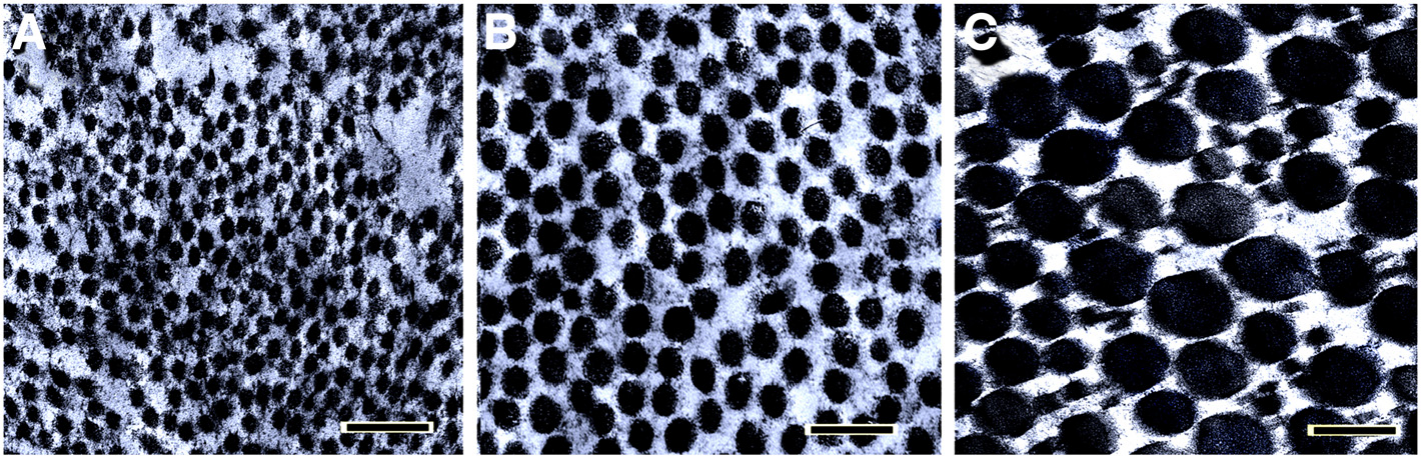
**Ultrastructural findings: A**) Injured tendon of a 28 DPI rabbit. The small sized collagen fibrils are not differentiated and are unimodally distributed. **B**) Injured tendon of an 84 DPI rabbit. The collagen fibrils diameters are larger than those of the 28 DPI rabbits and show a bimodal distribution. However, they are much smaller than those of the normal contralateral tendons. **C**) Normal contra lateral tendon: The collagen fibrils show a multimodal distribution pattern and collagen fibrils of different sizes are seen in this section (Scale bar = 360 nm).

However, the number of collagen fibrils and fibrillogenesis on day 84 post-injury was significantly less (*P = 0.004*), but the fibrils' diameter (*P = 0.001*) and density (*P = 0.004*) were significantly greater than the lesions of the 28 days post-injury (Figure 6, Table 2). At this stage, the new, regenerated collagen fibrils were differentiated into two populations of small fibrils of 33-64 nm and relatively large new regenerated fibrils of 65-102 nm, and the mean fibril diameter of these two categories was 51.12 ± 4.11 nm. This bimodal collagen fibrils pattern showed a proper alignment along the longitudinal axis of the tendon. Despite relative differentiation and enlargement of the collagen fibrils of the injured tendon on day 84 post-injury, they were still significantly smaller in diameter (*P = 0.001*) and density (*P = 0.001*) than those of their normal contra-lateral tendons.

Compared to the tenoblasts of the 28 days post injury lesions that were granulated and contained more rough endoplasmic reticulum, mitochondria, golgi apparatus, lysosomes, mitotic indices and collagen production capability in their cytoplasm, the tenoblasts and tenocytes of the 84 days post-injury lesions were more mature, contained lower amounts of cytoplasmic granules, rarely showed collagen production capacity, and many of them were almost comparable to those of the normal contra-lateral tenocytes (Table 2).

While the elastic fibers of the 28 days post-injury lesions were immature and consisted solely of parallel microfibrils, they were more mature on day 84 post-injury and a dense matrix began to be deposited from the center of the fiber and, in some instances, covered the central microfibrils. However, there was no significant difference in the number of elastic fibers on 28 and 84 days post-injury.

### Biomechanical findings

All samples failed from the mid part of the tendon during the tensile testing. The ultimate strength of the injured tendons on day 28 was significantly inferior to those of day 84 post-injury (*P = 0.001*) and they were 44.08% of their normal contralateral tendon (*P = 0.001*). Although, the ultimate strength of the injured tendons of the 84 days post injury animals showed 25.0% improvement compared to the 28 days post-injury ones, the ultimate strength of the injured tendons on day 84 post injury was still significantly inferior to their normal contra-lateral normal tendons (*P = 0.003*) and was only 59.1% of the normal tendon.

**Figure 7:**
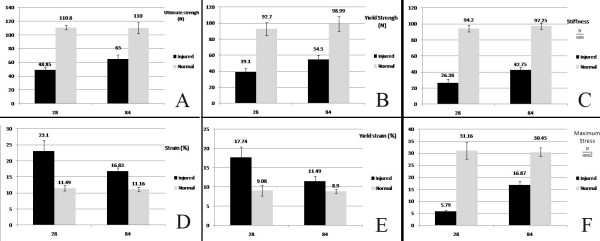
**Biomechanical properties: A**) Ultimate Strength, **B**) Yield strength, **C**) Stiffness, **D**) Ultimate strain, **E**) Yield strain, and **F**) Maximum stress of the injured tendon and their normal contralaterals of the animals of 28 DPI are compared with those of 84 DPI.

In addition, the yield strength, stiffness, ultimate strain, yield strain and stress of the injured tendons of the day 28 post-injury rabbits were significantly inferior to their normal contra-laterals and the injured tendons on day 84 post-injury respectively (for all *P = 0.002*). However, on day 84 post injury, these biomechanical parameters were still significantly inferior to their normal contra-lateral tendons (for all *P = 0.002*) (Figure 7). The maximum stress of the injured tendon showed the most significant improvement by time from day 28 to day 84 post-injury and increased from 18.6% of their normal contra-lateral on day 28 to 55.4% on day 84 post-injury. However, the ultimate stress of the injured tendons of the 84 day post-injury rabbits was still significantly inferior to their normal contra-lateral tendons.

### Dry matter

On day 28 post-injury, the amount of dry weight content of the injured tendon was significantly inferior to those of the 84 day post-injury (*P = 0.001*) and the injured tendons of both groups were still significantly inferior to their normal contra-lateral normal tendons respectively (*P = 0.001*, *P = 0.015*) (Figure 2B).

## Discussion

The results of the present study demonstrated that while the surgical protocol and time of immobilization was effective in improving the structural and physical characteristics of the experimentally induced transverse section SDFT after 12 weeks post-injury, it strongly elucidated that the healing of the sharp ruptured tendons is quite slow and, despite intensive remodeling over the following months, complete regeneration of the tendon was not achieved.

Normal tendon is presented as an array of parallel waveform fibers and tenoblasts [12,17]. After injury, collagen fibrils and fibroblasts are laid down in a random pattern without a preferred orientation, and no obvious waveform pattern ("crimp") can be seen [9,14]. Random orientation of the collagen fibers and tenoblasts at 28 days post injury, with little signs of crimp formation and high degree of cellularity consisting of numerous immature fibroblasts, lymphocytes, plasma cells and macrophages together with a homogenous population of small-sized, new regenerated collagen fibrils of 33-64 nm, resulted in a lower biomechanical property wound at this stage. In addition, low echogenicity with amputated view on ultrasonography with higher water content and higher tendon diameter of the injured tissue at 28 days post injury are other reasons why the biomechanical performance is still low at this stage of healing. The newly regenerated collagen fibrils are mostly of type III collagen fibrils, which is considered a weak and embryonated type of collagen [7,27], as they do not have axial periodicity and are not able to resist violent physical activities or higher biomechanical loads [1,8,28]. The ultimate strength of the injured tendons at this stage was 44.0% of their normal contra-laterals. Possibly, part of the biomechanical property of the injured tendon may still be due to the presence of the suture material that made part of their biomechanical performance at this stage. It has been stated that the suture material has an important role in tendon stability in the earlier stages of healing [14,17,18], and it is reported that after interosseous and extraosseous flexor tendon repair following tenotomy in rabbits, at biomechanical testing, 38 of the 40 injured tendons failed at the suture site [29].

At the later stages of healing, the edema diminishes, the tenoblasts start to mature to tenocytes and their number decreases [12,14]. Corresponding to a diminution in the number of tenoblasts, the collagen synthesis and degradation reaches equilibrium between 3 and 6 weeks after injury, the immature type III collagen decreases, the mature type I enhances, and the total collagen content becomes stable [11,30,31]. The newly formed tissue starts to mature, and the collagen fibrils are increasingly orientated along the direction of force through the tendon [9,14]. At the earlier stages of healing, the unorganized tissue lacks crimp pattern [1,27,28], but as the collagen fibrils are organized, they are bundled into large fibers that are evident throughout the tendon under light and polarized microscopes as a crimped pattern facilitating 1-3% elongation of the tendon [27]. This elongation of the individual fibers serves to buffer the tendon from sudden mechanical loading [32]. Interactions between collagen fibrils at the macromolecular level leading to the fiber unit architecture dictate the strength of the tendon [6,27,33]. Improvement in the alignment of the collagen fibers and tissue maturation together with an increase in the diameter and cross-linking of the collagen fibrils clearly explains why the biomechanical properties and physical performance of the injured tendons were more advanced at 84 days post injury compared to those of the 28 days post-injury.

Despite the relative improvement in the structural and biomechanical indices on day 84 post injury, compared to those of day 28 post-injury, the hierarchical organization and physical characteristics of the injured tendons were still significantly inferior to their normal tendons at this stage. The morphological and biomechanical findings of the present study showed that the remodeling phase of the tendon healing is possibly extremely prolonged and the area remains abnormal in several histological, ultrastructural, biomechanical and biochemical characteristics for a long period after the original injury is induced.

Maturation and differentiation of the collagen fibrils in the wound area is much slower than in the tendon of the normal growing animals [26]. It has been reported that the mean diameter of the collagen fibrils of a normal SDFT of a 28 day-old rabbit was 38.1 ± 2.3 nm, however, their diameter increased very quickly thereafter, so that the collagen fibrils of 162 nm and mean diameter of 124.5 ± 6.1 nm were seen in the 112 day-old rabbits [26], while in the present study, compared to the mean diameter of 193.3 ± 2.5 nm in the normal contra-lateral tendons, the collagen fibrils diameter of the injured area of the tendons at 84 days post-injury was 51.1 ± 4.1 nm. However, other tissue hierarchical organization was partly retrieved at this stage and most of the mesenchymal cells were mature tenoblasts. Nevertheless, the tissue was hypercellular and its percentage dry weight content was also significantly inferior to those of their normal contra-lateral tendons. Concomitant with the structural insufficiencies, the biomechanical properties of the injured tendons were not comparable to those of their normal contra-lateral ones so that the ultimate strength of the injured tendons on day 84 post-injury were only 59.1% of their normal contra-lateral tendons (*P =0.003*). In addition, the yield strength, stiffness, ultimate strain, yield strain and maximum stress of the injured tendons on day 84 post-injury were still significantly inferior to their normal contra-laterals (*P=0.002*) at this stage. Possibly, the ultimate strength on the earlier stages of healing in the present study is mostly dependent upon the fibrillogenesis which is demonstrated as the presence of numerous small regenerated collagen fibrils in the lesion, while at the later stages of healing the increase in the diameter of the fibrils and improvement in the collagen cross-linking together with tissue alignment are more important in gaining advanced biomechanical performance at this stage.

Potentially one of the blind points of the present study could be the time of the investigation.

However, if the experiment were to be continued for a longer time, the small collagen fibrils may enlarge and differentiate to a multimodal distribution pattern. This structural improvement could enhance the physical characteristics of the tendon at later stages of healing. In addition, determination of some of the biochemical criteria such as collagen type III and matrix metalloproteinases could also be helpful in explanation of various stages of tendon healing.

Therefore, this study could strongly demonstrate that the healing of the transverse sectioned tendons is substantially slow and, despite intensive remodeling over the following months, complete regeneration of the tendon is not achieved for a long time. The tissue replacing the defect remains hypercellular and the diameter of the collagen fibrils is altered, favoring small-diameter embryonic fibrils, and the biomechanical strength is significantly lower than the normal tendon. Tendon healing, even when successful, does not result in normal tendon, however, the biomechanical performance is possibly enough for routine life locomotion activities. Longer term ultrastructural, biochemical, molecular and biomechanical studies could demonstrate whether the morphological, physical and metabolic activities of the injured tendons finally coincide those of the normal contra-lateral tendons or not.

## Competing interests

The authors declare that they have no competing interests.

## Authors’ contributions

The authors have equal contributions in all parts of the study.
